# Clinical feasibility of interactive motion-controlled games for stroke rehabilitation

**DOI:** 10.1186/s12984-015-0057-x

**Published:** 2015-08-02

**Authors:** Kelly J. Bower, Julie Louie, Yoseph Landesrocha, Paul Seedy, Alexandra Gorelik, Julie Bernhardt

**Affiliations:** Department of Physiotherapy, Royal Melbourne Hospital, Melbourne, VIC Australia; The Florey Institute of Neuroscience and Mental Health, University of Melbourne, Melbourne, VIC Australia; Australian Catholic University, School of Exercise Science, Melbourne, VIC Australia; Current Circus, Melbourne, VIC Australia; Royal Melbourne Hospital, Melbourne Epicentre, Melbourne, VIC Australia

## Abstract

**Background:**

Active gaming technologies, including the Nintendo Wii and Xbox Kinect, have become increasingly popular for use in stroke rehabilitation. However, these systems are not specifically designed for this purpose and have limitations. The aim of this study was to investigate the feasibility of using a suite of motion-controlled games in individuals with stroke undergoing rehabilitation.

**Methods:**

Four games, which utilised a depth-sensing camera (PrimeSense), were developed and tested. The games could be played in a seated or standing position. Three games were controlled by movement of the torso and one by upper limb movement. Phase 1 involved consecutive recruitment of 40 individuals with stroke who were able to sit unsupported. Participants were randomly assigned to trial one game during a single session. Sixteen individuals from Phase 1 were recruited to Phase 2. These participants were randomly assigned to an intervention or control group. Intervention participants performed an additional eight sessions over four weeks using all four game activities. Feasibility was assessed by examining recruitment, adherence, acceptability and safety in both phases of the study.

**Results:**

Forty individuals (mean age 63 years) completed Phase 1, with an average session time of 34 min. The majority of Phase 1 participants reported the session to be enjoyable (93 %), helpful (80 %) and something they would like to include in their therapy (88 %). Sixteen individuals (mean age 61 years) took part in Phase 2, with an average of seven 26-min sessions over four weeks. Reported acceptability was high for the intervention group and improvements over time were seen in several functional outcome measures. There were no serious adverse safety events reported in either phase of the study; however, a number of participants reported minor increases in pain.

**Conclusions:**

A post-stroke intervention using interactive motion-controlled games shows promise as a feasible and potentially effective treatment approach. This paper presents important recommendations for future game development and research to further explore long-term adherence, acceptability, safety and efficacy.

**Trial registration:**

Australian and New Zealand Clinical Trials Registry (ACTRN12613000220763)

## Background

Stroke is a leading cause of disability world-wide [1]. Common stroke-related impairments, such as loss of strength, sensation and coordination, lead to difficulties in walking [2], balance [3], and upper limb function [4]. This can have a significant impact on an individual’s independence, safety and quality of life [5, 6]. Therefore, the implementation of effective interventions to optimise recovery is critical.

Physical therapy has been shown to aid recovery after stroke [7–9]. A recent systematic review and meta-analysis [7] demonstrates strong evidence in favour of physical therapy interventions for gait training, balance, upper limb function, activities of daily living and physical fitness. Although the optimal dosage and type of activity for improving outcomes after stroke remains unclear, research generally favours intensive and repetitive task-specific training [7, 9]. However, barriers such as resource limitations, access to therapy, patient motivation and safety may contribute to the low levels of physical activity observed in hospital settings [10] and reduce long-term adherence to exercise regimes.

Motion-controlled video games, including the Nintendo Wii and Xbox Kinect, have become an increasingly common adjunct to physical therapy and show potential as effective and feasible post-stroke treatment options [11, 12]. The engaging nature of a game-based approach may serve to increase motivation and repetitive practice [11, 13, 14]. The variety of activities presented can allow for the practice of a range of physically and cognitively challenging tasks [14]. Furthermore, the feedback provided by gaming systems may enhance motor learning and motivation [15], and allow for objective monitoring of performance over time. Although few high-quality studies have been published to date, Nintendo Wii-based training after stroke has demonstrated improvements in upper limb function [11, 16, 17] and balance [18, 19], with high levels of acceptability and minimal safety concerns. The more recently released Xbox Kinect, which uses a three-dimensional (3D) depth-sensing camera, has not been extensively studied. One trial found improvements with additional upper limb training after stroke [12], and studies in other neurological populations have demonstrated positive preliminary findings [20, 21].

Despite the potential utility of consumer video game systems for stroke rehabilitation, a number of limitations have been highlighted. Games designed for the general population can be too challenging or inappropriate for people with physical and cognitive deficits [22–24]. For example, individuals with stroke may struggle with manipulating controllers (e.g. Nintendo Wii remote) [24] and responding to activities that are fast and visually complex (e.g. Kinect Sports games) [25]. The difficulty levels and control of the games are often not readily adjustable (e.g. calibrating the Wii Balance Board for individuals with asymmetries) and the tasks may lack functional relevance [24]. Furthermore, the feedback and scoring provided can be negative and frustrating for the user [24, 25]. Therapists have highlighted desirable features of video games as being able to record meaningful data, include a variety of games, provide positive feedback and have the ability to grade the task difficulty [26]. In response to some of these limitations, there has been an emergence of research and development of games specifically designed for rehabilitation using components of these systems [27–29]. However, these approaches have largely not progressed beyond initial development phases with little testing undertaken in clinical populations and settings.

The aims of this study were therefore to: 1) develop a suite of gaming activities using a low-cost depth-sensing camera suitable for use with people affected by stroke undergoing rehabilitation; 2) investigate the usability, acceptability and safety of these activities across a broad range of people with stroke within a clinical rehabilitation setting; and 3) explore changes in clinical outcomes in people exposed to additional game-based exercises compared with standard care. It was hypothesised that: 1) a broad range of people with stroke would be capable of using the developed games; 2) the majority of participants would find the games enjoyable, helpful for their recovery and something they would like to continue using; 3) there would be few safety concerns. We also aimed to examine changes in functional outcome measures over time to inform future efficacy studies.

## Methods

### Game development

The software for the four games used for testing was developed through collaboration between researchers, physiotherapy clinicians and a game development company, Current Circus (Melbourne, Australia). Games were selected by the clinicians from a range of prototypes already under development and modifications were made prior to implementation in Phase 1 of the study. These games used a PrimeSense ‘Carmine’ depth camera (PS1080), which was connected via USB to a laptop computer with graphics displayed on a television screen. The camera uses a 3D depth sensor, which is the same technology used in the Microsoft Kinect for Xbox360 and Kinect for Windows V1, enabling the user to interact with the game environment without the need for controllers or body-worn sensors. The camera is able to detect a range of 0.8 to 3.5 m, with an ideal distance of 2.5 m. The camera’s runtime software contains image processing algorithms for the purpose of identifying human shapes. Following an auto-calibration process, ideally with the user standing facing the camera, a hierarchy of skeleton joints is constructed. It is able to track multiple users; however, the software was limited to a single user for our purposes. The skeleton data can be tracked while the user is in a seated or standing position. The game activities were designed to minimise inaccuracies with skeleton tracking and to simultaneously trigger the desired movements of the rehabilitation exercises. Participants were able to interact with the games whilst having physical assistance from a therapist or using any wheelchair or gait aid if positioned behind or to their side. The software was developed with a Unity3D game engine using runtime libraries ‘OpenNI’ and ‘NITE’ developed by PrimeSense.

**Fig. 1:**
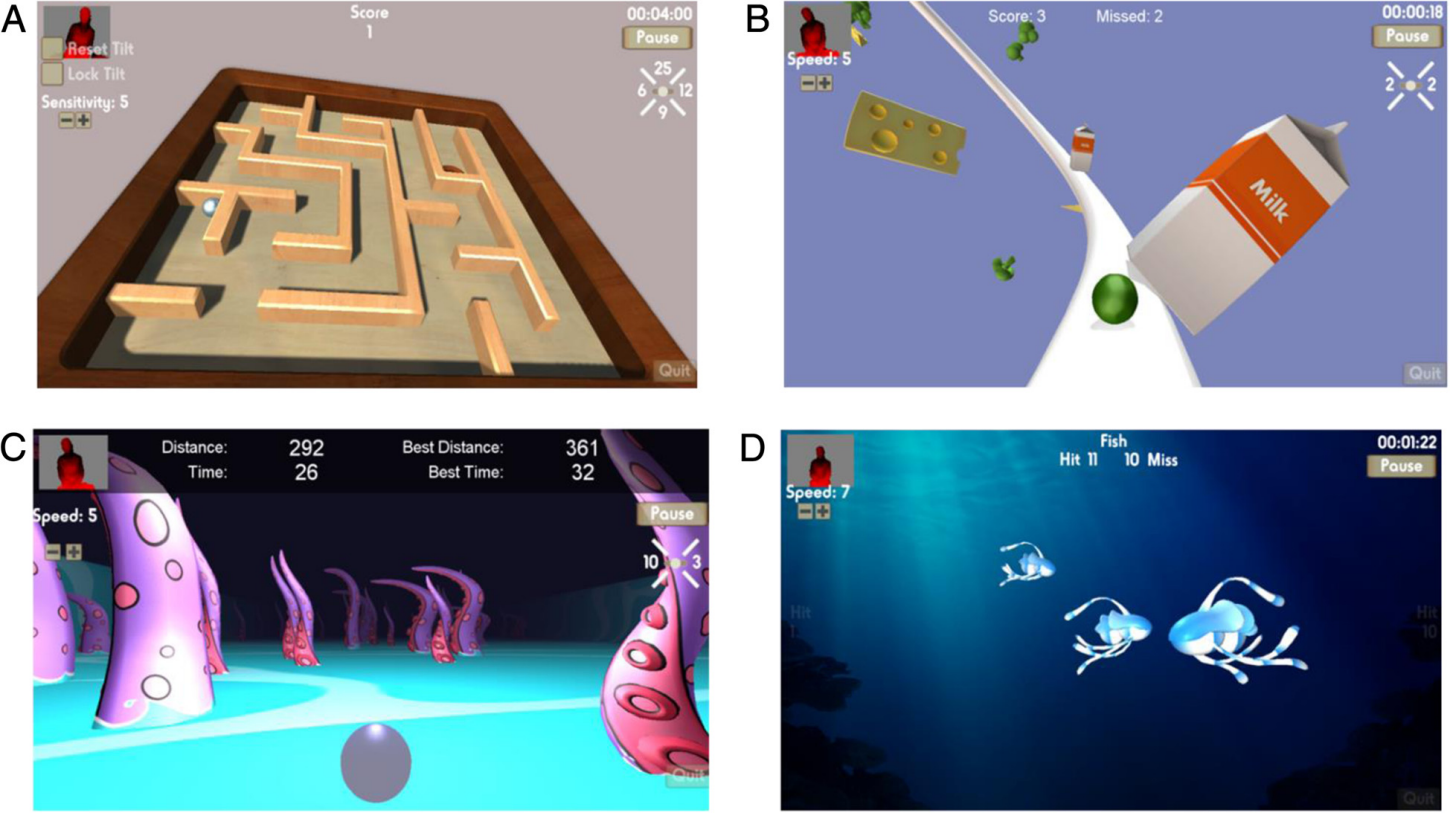
Screen shots of the four game activities. *Legend:* **a**. Ball Maze **b**. Fridge Frenzy **c**. Tentacle Dash **d**. Bubble Fish

The games were developed to encourage dynamic balance and upper limb activities, and be adaptable to users with different levels of balance, motor control and perceptual problems commonly found after stroke. Three of the games involved weight-shifting movements of the torso and one game encouraged upper limb activity. Screen shots of the games can be seen in Fig. 1 and are described below.

#### (1) Ball Maze

Motion of the shoulders and hips was tracked. Leaning movements of the torso (forward, backward, left and right) resulted in tilting of the maze board. The aim was to guide the ball around the maze into the hole. The number of movements in each direction was automatically recorded by the program. Points were awarded each time a ball was successfully manoeuvred into the hole.

#### (2) Fridge Frenzy

Motion of the shoulders and hips was tracked. Lateral flexion movements of the torso resulted in side-to-side movement of the ball as it progressed along a track, with the aim to hit the milk cartons. The number of left and right movements was recorded by the program. Points were displayed for the number of hits and misses.

#### (3) Tentacle Dash

Motion of the shoulders and hips was tracked. Movement of the torso from the initial midline position, through leaning or side-stepping, resulted in side-to-side movement of the ball as it progressed forwards, with the aim of avoiding hitting the tentacles. If a tentacle was hit the game started again. The distance travelled and time taken was displayed.

#### (4) Bubble Fish

Motion of the wrist joint relative to the shoulder was tracked. Movement of the arm resulted in bubbles shooting forwards in different directions, with the aim to hit the fish. The fish moved in from both the left and right sides of the screen and at different depths from the user. Points were displayed for the number of fish hit and missed and whether these were from the left or right side of the screen.

A number of attributes were considered when developing the games to allow for maximal participant inclusion even at very early stages of rehabilitation following stroke. All of the games allowed the user to interact in a seated or standing posture and each had 10 levels of difficulty. With the exception of ‘Ball Maze’, difficulty levels were based on required response speeds to moving virtual objects. Difficulty in ‘Ball Maze’ was adjusted based on the sensitivity of the response of the board tilting to the individual’s body movement (i.e. larger movements of the torso at lower levels, versus smaller and more controlled movements at higher levels). Visual distractions within the games were minimised as this was seen as potentially too challenging, particularly for individuals with cognitive and perceptual post-stroke deficits. However, apart from ‘Ball Maze’ the games inherently became more visually challenging as users were required to respond more quickly to visual stimuli. Virtual objects in ‘Tentacle Dash’ and ‘Bubble Fish’ (i.e. tentacles and fish) were randomly generated at the beginning of each game so that the movement was not predictable. Conversely, ‘Fridge Frenzy’ had a set pattern of objects over a period of time that looped and ‘Ball Maze’ had four variations based on the orientation of the maze board that were randomly presented.

Features were built into the games to allow for objective monitoring and feedback on performance. All four games had scoring and time counts as previously described. Additionally, a small depth representation of the user could be seen in the upper left corner (Fig. 1). This allowed immediate feedback on movement; however, given the focus on the game activity it was unlikely to be used as a key feedback mechanism. Simple auditory feedback was provided in each game in response to either successful or unsuccessful movements or ‘hits’.

### Phase 1: Initial feasibility testing

Forty adults with stroke were consecutively recruited from inpatient and outpatient services at a single rehabilitation facility in Melbourne, Australia, from August 2012 to April 2013. Eligible participants were adults with haemorrhagic or ischaemic stroke who were able to sit unsupported for greater than 10 s (Motor Assessment Scale - Sitting Balance ≥ 2 [30]). Individuals were excluded if they had severe dysphasia, significant cognitive deficits (Mini-Mental State Examination < 20 [31]), other medical conditions (e.g. progressive neurological condition, severe arthritis, unstable heart condition) impacting on their ability to participate in the study, or visual problems such that they weren’t able to adequately see the games when displayed on the television screen. There were no restrictions in regard to the length of time since stroke. All participants were receiving concurrent therapy, at various intensities, either though the inpatient or outpatient rehabilitation services. Ethical approval was obtained from the Melbourne Health Research Ethics Committee (ID: 2011.210) and written informed consent obtained from all participants.

Demographic information and stroke details were collected at baseline, in addition to the Functional Independence Measure (transfers, walking and stairs) [32], Motor Assessment Scale [30] and the Functional Reach [33]. Feasibility outcomes addressed: 1) recruitment rate and willingness to participate; 2) adherence, through documentation of session attendance and length; 3) acceptability, using 5-point Likert scales [34] to rate enjoyment (from 1: “really didn’t enjoy” to 5: “really enjoyed” in response to “I enjoyed my treatment session”) and perceived helpfulness (from 1: “really not helpful” to 5: “really helpful” in response to “I thought my session today was helpful for my recovery”), and ‘yes/no’ response for continued use of the game; and 4) safety, through documentation of any adverse events, including pre- and post-session ratings of pain and fatigue using an 11-point vertical visual analogue scale (VAS) [35, 36] and a post-session rating of perceived exertion using the Borg scale (rated 6–20) [37]. Serious adverse events were classified as falls or any safety events requiring medical attention. Furthermore, any subjective reports of other symptoms were recorded. Finally, participants were asked to give feedback during each session to provide further information in regard to acceptability and suggestions for improvements and this was recorded by the treating therapist.

Stratified block randomisation was used to allocate each participant to one of the four gaming activities. Each participant completed one gaming activity during a single session, under the supervision of a physiotherapist. The protocol involved participants completing all 10 levels of the game, first in sitting, then in standing as able, with each level lasting approximately one minute.

### Phase 2: Pilot randomised controlled trial

Of the 40 participants in Phase 1, 16 were consecutively recruited to participate in Phase 2 of the study. Recruitment for Phase 2 commenced after 15 participants had completed Phase 1 of the study, with all participants from this time point onwards invited to take part. Eligibility criteria were identical to the Phase 1 participants. Participants in Phase 2 were randomly assigned to an intervention or control group. The intervention group (*n* = 8) completed eight 40 min sessions over four weeks, in addition to their standard inpatient or outpatient therapy. During the first two sessions participants used all four gaming activities. In the subsequent sessions they were able to choose which activities they wished to undertake. Participants in the control group (*n* = 8) continued with standard care only, consisting of inpatient or outpatient therapy.

Feasibility data collected in Phase 2 were identical to Phase 1; however, in addition to the documentation of informal feedback during the sessions, participants in the intervention group were specifically asked ‘What three things did you like the most?’ and ‘What things would you change?’ at the completion of their study participation. Furthermore, the following functional outcomes were assessed at baseline and four weeks for Phase 2 participants: Functional Independence Measure (transfers, walking and stairs) [32], Motor Assessment Scale [30], Functional Reach [33], Step Test [38], and 6-min walk test [39]. An assessor, blinded to group allocation, collected the post-intervention outcome data. Both the intervention and control groups continued their usual therapy sessions during their study participation. This typically consisted of one to three hours of physiotherapy and occupational therapy five days per week for inpatients, and one to two therapy sessions per week for outpatients. Participant opinions and feedback regarding their usual care were not sought.

### Statistical analysis

Participant characteristics and functional outcome measures at baseline for both Phase 1 and Phase 2 participants were summarised using descriptive statistics. The normality of data distribution was evaluated using Shapiro-Wilk tests. One-way analysis of variance (ANOVA) or Kruskall-Wallis tests were used to assess baseline differences between the four groups in Phase 1. Independent t-tests, Mann–Whitney U tests or Chi square tests were used to assess differences in baseline characteristics between the two Phase 2 groups, and between the Phase 1 and Phase 2 groups.

Descriptive statistics were used to summarise session length, time spent in each game activity, standing versus sitting times, and difficulty levels reached. Likert ratings of enjoyment and perceived helpfulness were reported descriptively for participants in both phases. Kruskall-Wallis tests were used to assess differences in acceptability ratings between the four Phase 1 groups. Participant feedback was compiled by a member of the research team and key themes and comments described.

Changes in pain and fatigue were reported descriptively for participants in both phases. One-way ANOVA were used to examine differences in changes in pain and fatigue within and between the four Phase 1 groups. Borg ratings of perceived exertion were compared between Phase 1 groups using a Kruskal-Wallis test, and between Phase 1 and Phase 2 groups using a Mann–Whitney *U* test.

The number of usual therapy sessions received by the intervention and control group in Phase 2 was compared using independent t-tests. Phase 2 functional outcomes were presented descriptively, including within-group change scores (mean (SD)) and between-group differences (mean 95 % CI). Within-group changes were evaluated using Wilcoxon signed-rank tests and between-group differences at Week 4 were assessed using Mann–Whitney U tests.

## Results

### Recruitment and participant details

**Fig. 2:**
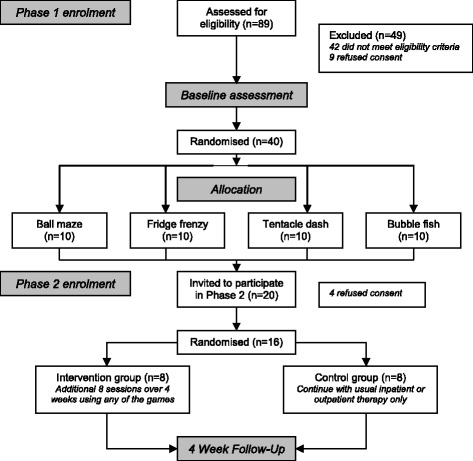
Study flow diagram

Phase 1: Forty of 89 individuals screened agreed to take part in Phase 1 of the study; 42 were ineligible and seven declined consent (Fig. 2). As people with stroke from slow-stream rehabilitation wards were also screened, the primary reasons for exclusion were due to significant cognitive or physical deficits (i.e. unable to sit unsupported or adequately follow instructions). Phase 1 participants were a mean age of 63.1 years, with a median time since stroke of 5.5 weeks (Table 1). Mini-Mental State Examination scores ranged from 20 to 30 and Motor Assessment Scale scores ranged from 9 to 48. No significant differences between the four groups within Phase 1 were observed.

**Table 1:** Participant baseline characteristics

Demographics, stroke details and functional status	Phase 1	Phase 2	
	(*n* = 40)	Intervention (*n* = 8)	Control (*n* = 8)
Age, mean (SD), years	63.1 (15.4)	60.8 (16.1)	60.9 (14.0)
Male : Female	27:13	5:3	6:2
Inpatient : Outpatient	29:11	5:3	4:4
Time since stroke, median (IQR), weeks	5.5 (2.5-23.4)	12.8 (3.9-137.8)	24.7 (5.8-51.1)
Infarct : Haemorrhage	31:9	4:4	6:2
Left : Right side of lesion	16:24	3:5	3:5
Mini-Mental State Examination, mean (SD), /30	26.3 (3.2)	26.6 (3.2)	24.0 (3.1)
Functional Independence Measure, mean (SD), /7			
Transfers	6 (4–6)	6 (4–6)	5.5 (4.3-6)
Walking	5 (2–6)	5.5 (2.5-6)	5 (2–6)
Stairs	5 (1–6)	5.5 (1.8-6)	5 (1–6)
Motor Assessment Scale, median (IQR), /48	36 (27–44)	29 (24–36)	36 (25–39)
Functional Reach, mean (SD), cm	26.1 (9.0)	24.0 (8.0)	25.4 (8.9)

**Table 2:** Phase 2 functional outcomes

Outcome measures^a^	Week 0		Week 4		Within-group difference (Week 4 – Week 0)^a^		Between-group difference (Mean 95 % CI)
	Intervention	Control	Intervention	Control	Intervention	Control	Intervention - Control
FIM transfers, /7	6.0 (4.0-6.0)	5.5 (4.3-6.0)	6.5 (6.0-7.0)	6.0 (5.0-7.0)	1.0 (1.1)*	0.6 (1.1)	0.4 (−0.8 to 1.6)
FIM mobility, /7	5.5 (2.5-6.0)	5.0 (2.0-6.0)	6.5 (6.0-7.0)	6.0 (2.8-7.0)	1.8 (1.7)*	1.0 (1.7)	0.8 (−1.0 to 2.6)
FIM stairs, /7	5.5 (1.8-6.0)	5.0 (1.0-6.0)	6.0 (4.3-6.0)	5.0 (2.0-6.0)	0.6 (1.4)	0.5 (1.9)	0.1 (−1.7 to 1.9)
Motor Assessment Scale, /48	29.0 (24.0-36.0)	35.5 (24.8-39.0)	33.5 (26.3-39.8)	35.5 (23.5-44.8)	2.4 (4.7)	2.4 (5.6)	0 (−5.5 to 5.5)
Functional Reach, cm *Unable to do, N (%)* ^*b*^	24.0 (8.0) *1 (12.5 %)*	25.4 (8.9) *1 (12.5 %)*	26.3 (8.3) *1 (12.5 %)*	28.3 (14.0) *1 (12.5 %)*	2.3 (8.4)	3.8 (9.1)	−1.5 (−10.9 to 7.8)
Step Test (affected), number of steps in 15 s *Unable to do, N (%)* ^*b*^	0 (0–9.8) *5 (62.5 %)*	8.0 (0–11.0) *3 (37.5 %)*	2.5 (0–13.0) *4 (50 %)*	1.0 (0–8.3) *4 (50 %)*	1.6 (5.0)	−2.4 (5.3)	4.0 (−1.5 to 9.5)
Step Test (unaffected), number of steps in 15 s *Unable to do, N (%)* ^*b*^	2.0 (0–10.3) *4 (50 %)*	6.0 (0–7.0) *3 (37.5 %)*	6.0 (0–11.5) *3 (37.5 %)*	2.5 (0–10.3) *4 (50 %)*	2.0 (4.0)	0 (5.8)	2.0 (−3.3 to 7.3)
6 min Walk Test, m *Unable to do, N (%)* ^*b*^	82 (0–248) *3 (37.5 %)*	95 (0–288) *3 (37.5 %)*	160 (110–276) *0*	274 (45–306) *1 (12.5 %)*	64.3 (69.4)*	75.1 (151.9)	−10.8 (−137.4 to 115.8)

Abbreviations: FIM, Functional Independence Measure; affected, affected leg in stance; unaffected, unaffected leg in stance during test

^a^Presented as mean (SD) or median (IQR); ^b^If unable to do, the score was recorded as zero

*Significant within-group difference *P* < 0.05 (Wilcoxon signed-rank test)

Phase 2: Twenty participants included in Phase 1 were consecutively invited to participate in Phase 2 and 16 accepted. Four Phase 1 participants declined to participate due to lack of interest, time commitments or discharge date from the inpatient ward occurring within the next four weeks. Phase 2 participants were a mean age of 60.8 years, with a median time since stroke of 18.5 weeks (Table 1). Mini-Mental State Examination scores ranged from 20 to 30 and Motor Assessment Scale scores ranged from 11 to 44. With the exception of time post-stroke (*P* = 0.04), Phase 2 participants were not significantly different to Phase 1 participants at baseline.

### Adherence

Phase 1: All 40 participants completed a single session using one of the four games. Mean (SD) session time was 33.6 (7.9) minutes. The full 10-level sitting and standing protocol was completed by 58 % (*n* = 23) of participants. Five participants in Phase 1 were unable to complete any game levels in standing. The mean (SD) percentage time spent in standing across all participants was 43 (16) %. Those who were unable to complete the full protocol tended to have a lower level of functional ability or became fatigued during the session. The mean (SD) time spent actively using the games within each session and the number of movement counts (where applicable) were as follows: ‘Ball Maze’ 22.2 (7.2) minutes and 466 (209) leaning movements of the torso in all four directions; ‘Fridge Frenzy’ 19.1 (4.0) minutes and 218 (85) leaning movements to the left and right; ‘Tentacle Dash’ 21.3 (10.6) minutes; and ‘Bubble Fish’ 18.9 (3.7) minutes.

Phase 2: Participants attended a mean (SD) of 7.3 (1.4) or 91 % of planned sessions with an average session time of 26.3 (9.3) minutes. One participant ceased participation after four sessions secondary to fatigue and neck pain. Phase 2 participants spent an average of 75 % of the time in standing, with two participants performing all activities in a standing position. Participants were allowed the freedom to choose which games they used in sessions 3 to 8. This percentage of utilisation was: ‘Ball Maze’ 29 %, ‘Fridge Frenzy’ 28 %, ‘Tentacle Dash’ 30 % and ‘Bubble Fish’ 13 % of total time. The median (range) maximal level of difficulty (out of 10) reached for each game was: ‘Ball Maze’ 7.5 (1–10), ‘Fridge Frenzy’ 8 (1–10), ‘Tentacle Dash’ 6 (1–10), and ‘Bubble Fish’ 3 (3–10), with *n* = 4, 3, 2 and 2 participants able to reach the maximal difficulty level for the four games respectively.

### Acceptability

Phase 1: The majority of participants reported the sessions to be enjoyable (92.5 % rated “enjoyed” or “really enjoyed” on the 5-point Likert scale) and felt the session was helpful for their recovery (80 % rated “helpful” or “really helpful”). One participant did not find the game-based session to be enjoyable or helpful, whereas others were neutral in their response. When asked whether they would like to continue the game intervention as part of their ongoing therapy, 87.5 % responded ‘Yes’. There were no significant differences in acceptability ratings of enjoyment (*P* = 0.74) or perceived helpfulness (*P* = 0.29) between the four games in Phase 1.

Phase 2: Six of the eight Phase 2 participants reported enjoying the game sessions and five felt the activities were helpful for their stroke recovery.

### Participant feedback

Feedback from participants was mainly related to enjoyment, perception of benefit, ease-of-use and suggestions for improvement.

Participants felt the games were a fun and novel way of performing exercise and appreciated the competitive element. *“It’s a bit of fun and something different”* (Tentacle Dash P36). *“I like the variety. It’s good to test your skills with something new”* (Phase 2 P16). *“I want to know if I’m the winner. That’s what happens - you become competitive”* (Tentacle Dash). However, others felt the games were quite monotonous and lacked interest. *“It’s a bit repetitive if you just keep doing this game”* (Fridge Frenzy P28). *“I never really liked games - it’s not for me”* (Ball Maze P39).

Comments were made in regard to perceived helpfulness of the games on both physical and cognitive abilities. *“It helped me move my arm, which I haven’t done in a long time. I’ve been scared to move it”* (Bubble Fish P35). *“This game is good for my memory - I have to think ahead where to move the ball”* (Phase 2 P12). Others did not feel the game activities were of benefit. *“I don’t understand how it would help. It would probably help for a younger person but not for me. I’m over 80. It’s hard to understand for elderly people”* (Tentacle Dash P8).

Participants commented on issues related to usability. Some found the games either too easy or too difficult. *“It’s pretty easy for me. I felt like I would perform the same whether I had a stroke or not”* (Ball Maze P30). *“It’s hard to get the coordination and speed of movement right”* (Fridge Frenzy P31). Others expressed frustration with the movement controls. *“See you hit them and nothing happens!”* (Bubble Fish P11). *“Sometimes it doesn’t move when you’re leaning”* (Tentacle Dash P26). Participants also commented on their improvements over time. *“I’ve started to plan ahead better and for look what’s coming”* (Phase 2 P8).

Finally, several suggestions were made for improvements to the games. Participants commented that more variety and challenge would be desirable. *“Make the games go faster - to level 15 or so - (as) I got used to it”* (Phase 2 P2). *“You could make it more colourful with more interesting things”* (Fridge Frenzy P37). It was also suggested that better feedback on scores would be helpful. *“I want the scoreboard to come up on the screen”* (Phase 2 P8).

### Safety

There were no falls or serious adverse events requiring medical attention during any of the Phase 1 or Phase 2 sessions. However, pain, which is common after stroke, was present prior to commencing the game-based session in 25 % of Phase 1 and 20.7 % of Phase 2 sessions.

Phase 1: At the end of Phase 1 there was no significant overall change in pain rating (mean (SD) of 0.4 (2.2), *P* = 0.27) compared with the pre-session score. However, changes in pain were reported in 42.5 % (*n* = 17) of participants. Pain increased in 12 participants (ranging from 1 to 8 points), while five participants reported improvements in pain (ranging from 1 to 5 points) following the game-based session. The rate of pain occurrence was, in general, evenly spread between the four game activities, with no significant difference between change in pain scores (*P* = 0.87).

Phase 2: Six of eight Phase 2 participants reported an increase in pain (ranging from 1 to 8 points) in 13 of 58 total sessions. Pain reductions were seen in 10 sessions (ranging from 1 to 2 points). The highest rating of increased pain was reported in the participant who discontinued the study after four sessions due to neck discomfort. Although these symptoms were likely exacerbated by study participation, they were also reported during their usual physiotherapy sessions. Furthermore, one participant in Phase 2 complained of dizziness, which increased by 2 to 3 points (on an 11-point scale) during each session and limited their study session duration. This dizziness was also reported during their usual physiotherapy sessions and was related to their type of stroke.

Overall pre to post-session fatigue in Phase 1 participants increased by a mean (SD) of 1.6 (2.4) on an 11-point VAS (*P* < 0.001). Fatigue increase (ranging from 1 to 8 points) occurred in 22 of 40 Phase 1 participants. Three participants reported a decrease in fatigue, ranging from 1 to 2 points. There was no significant difference between change in fatigue scores between the four game groups in Phase 1 (*P* = 0.41). Similarly, Phase 2 participants were found to have fatigue increases in 25 of the 58 total sessions (ranging from 1 to 5 points and reported by all eight participants), and decreases in three sessions (ranging from 1 to 3 points and reported by three individual participants). The post-session Borg rating of perceived exertion was a median (IQR) of 11.0 (9.5-13) in Phase 1, with no significant differences between the four groups (*P* = 0.45). Phase 2 participants reported a median (IQR) of 11.9 (8.9-13.1), which was not significantly different than Phase 1 ratings (*P* = 0.97).

### Phase 2 functional outcomes

Outcome data for Phase 2 are presented in Table 2. There were no significant between-group differences at baseline or at 4 weeks on any outcome measure. The intervention group improved significantly over time on several outcomes including FIM transfers (*P* = 0.04), FIM mobility (*P* = 0.03), and the 6-min walk test (*P* = 0.01). There were no significant within-group changes in the control group in any of the outcomes measures. A large number of participants were unable to perform the Step Test at either baseline or after 4 weeks (50 %; *n* = 8); and the 6-min walk test at baseline (37.5 %; *n* = 6). The number of usual therapy sessions (including physiotherapy, allied health assistant and exercise group sessions) received during the period of study participation did not significantly differ between the two Phase 2 groups (mean (SD) session number 15.5 (10.4) and 12.3 (10.5) in the intervention and control group, respectively; *P* = 0.54).

## Discussion

This study found that a treatment approach utilising 3D motion-tracking games was a feasible option for use in people with stroke, with high levels of acceptability. However, concerns in regard to safety and applicability across different functional levels require further exploration. Participant acceptability, in terms of enjoyment, perceived benefit for their stroke recovery, and desire for continued use, was relatively high. Participants were able to engage in repetitive physical activity without major safety concerns. However, there were a relatively large number of participants reporting minor increases in pain before and after the game-based sessions. More research is needed to explore the efficacy and longer-term feasibility of this approach.

This study aimed to develop games suitable for a broad range of people with stroke and sought to recruit participants who reflected this diversity. Although the heterogeneity of the study population may strengthen the generalisability of the findings, recruitment of a more targeted population may have resulted in different outcomes. As the games were designed to be applicable across a range of post-stroke abilities, it was therefore not expected that all levels of the games could be completed by all participants. Indeed, the inclusion of individuals with poor physical function impacted on the ability of these participants to complete the full study protocol. Several participants were unable to partake in the higher game levels and could not perform the activities in a standing position. The relatively prolonged concentration and attention required, as well as the repetition of one type of physical task, may have also been too demanding for some participants. This is consistent with findings presented by Galna et al. (2014), who found that people with Parkinson’s disease struggled with some of the physical and cognitive challenges presented in their Kinect-based game intervention [28]. However, participants with significant impairments in the current study were able to successfully engage in at least the lower levels of the games, and while it was challenging, it would be expected to become less so as they improved.

Conversely, several highly functioning participants felt they weren’t being challenged enough by the games and this may have led to boredom with repetitive practice. Arguably, these participants may have been better suited to using commercial games such as the Nintendo Wii, or other systems which provide a greater level of physical or cognitive challenge. However, the study protocol used allowed the researchers to develop a clearer understanding of the likely response and progression that could be expected in people with different levels of post-stroke disability to these games.

Acceptability of the game-based intervention was generally high. This is consistent with previous studies of Wii-based interventions following stroke [17, 18, 40] and Kinect-based interventions in other neurological populations [28, 41]. Although most participants found the games to be enjoyable and potentially helpful for their recovery, it would be valuable to investigate longer-term acceptability and adherence. Indeed most previous research has evaluated game-based interventions of two to six weeks duration in people after stroke [12, 16–18, 40]. Acceptability in this study appeared to be lower in the most highly or poorly functioning participants. Acceptability may have been affected by the study design, particularly in Phase 1, as participants were asked to perform one game activity only and progress through all levels of difficulty in seated and standing positions. Greater individual adaptation was included in Phase 2 from sessions 3 to 8, where participants were asked to choose, in collaboration with the therapist, the number of games, time spent on each game and level of difficulty. Acceptability of the game-based intervention may have been enhanced through the provision of a larger range of activities, more engaging and varied interfaces, aligning the tasks more closely to participant goals, and enabling individuals to work at their optimal level of challenge.

No major adverse safety events occurred within the study sessions; however, the incidences of pain reported in this study imply that this should be carefully monitored and activities adapted where appropriate. It is difficult to determine whether the pain reported in this study was significantly different from what was experienced during participants’ usual therapy sessions as this was not recorded. Furthermore, the incidence of pain in post-stroke populations has been reported as high [42] and the validity of using pain scales in this population has been questioned [36]. However, given the repetitive nature of the activities performed, and the possibility of individuals ignoring pain symptoms due to high levels of engagement and motivation [43], these findings suggest caution and close monitoring during implementation. It may also be advisable to increase the variability and graduate the intensity and duration of practice.

Fatigue scores varied considerably in this study; however, the overall mean increase was below what may be considered as clinically significant [35]. Calculated from the findings by Tseng et al. 2010, the standard error of measurement (SEM) and minimal detectable change (MDC) scores for post-exercise fatigue change using a VAS in individuals with stroke are estimated as 1.2 and 3.4 points respectively. Although the fatigue scale used was not able to differentiate the type of fatigue reported, a number of participants commented on feeling greater cognitive than physical fatigue. This was also reflected in the informal participant feedback and comments during the game-based sessions and would be an interesting area for further research. Perceived exertion in both phases of the study was rated as “fairly light”. This finding is consistent with previous research investigating the use of the Nintendo Wii in individuals with stroke [16, 18]. The level of fatigue and exertion experienced may affect an individual’s focus of attention and subsequent motor learning. It has been suggested that an external focus of attention (such as that encouraged by video game use) is beneficial for motor learning and also may reduce internal sensations of fatigue and exertion; however, as exertion increases it will tend to dominate an individual’s attention [44]. The clinical significance of increases in fatigue or exertion and the subsequent impact on an individual’s attention and motor learning is relatively unexplored and an important area for future research.

This feasibility study was not designed to detect significant changes in functional outcomes, rather we wanted to explore the utility of a range of measures related to the trained task (i.e. primarily standing based activity and balance). However, the findings indicated greater changes in mobility outcomes including the FIM (transfers and mobility) and the 6-min walk test in the intervention group. Although these findings must be interpreted with caution, the games could have resulted in improvements in these measures due to training which was primarily focused on weight-shifting and endurance in standing activities. Interestingly, the Functional Reach did not show significant changes despite the games challenging trunk control and upper extremity movement. This result may have been influenced by ceiling effects of this outcome measure. Single leg balance and upper limb activities were not highly trained by the game activities, which may be why other outcomes did not see significant changes.

Surprisingly, many of the outcomes did not significantly change within the groups over the four weeks despite participants also undertaking standard inpatient or outpatient therapy during that time. This may be explained by several factors. Phase 2 of the study recruited a relatively small number of participants with varying impairments and at different times following stroke. The type of training provided may not have provided an adequate level of challenge to allow for functional improvements in some participants. Concurrent therapy ranged from multiple sessions per day in more acute participants, to weekly in those who were in the chronic post-stroke phase. Although the usual therapy provided did not significantly differ between study groups, the type and amount of concurrent therapy received likely impacted on functional gains. The Phase 2 game-based intervention was relatively short and it has been suggested that at least 16 h of additional therapy is required to demonstrate functional gains after stroke [45]. Additionally, the type and amount of game-based activities varied between participants from sessions 3 to 8. However, it was noted that participants in Phase 2 completed on average an additional 20 min of standing activity during each 26 min treatment session. Therefore, this type of intervention shows potential as an effective means to increase engagement in physical activity, found to be particularly low in hospital settings following stroke [10]. Furthermore, consistent with previous research on game-based interventions [13], participants were seen to engage in a relatively high number of movement repetitions during each session.

Although the outcome measures were selected as potentially sensitive measures, which approximate the task demands of the game intervention, the use of other outcomes may have resulted in different findings. Several of the measures suffered from floor and ceiling effects, thereby reducing potential responsiveness within certain participant groups. For example, a large number of participants were unable to perform the 6-min walk test at baseline, and the Step Test at either baseline or after four weeks. Conversely, scores for the Functional Reach suffered from ceiling effects as they were generally within the range of normative values. Furthermore, separating the Functional Independence Measure into single item subcomponents has not been validated; however, assessing the full scale was not feasible for the purposes of this study. As the game-based intervention typically encouraged trunk and weight-shifting activities over a fixed base of support in a seated or standing position, alternative measures, such as the Trunk Impairment Scale [46], Fugl-Meyer Assessment [47] or Postural Assessment Scale for Stroke [48], may have been more responsive in lower functioning participants. A specific upper limb measure, such as the Wolf Motor Function Test [49], may also have been more appropriate for detecting any changes resulting from the upper limb training component. These would be recommended for future efficacy studies.

This feasibility study had a number of limitations. As the games were in the development phase, there were a restricted number of activities to choose from and a relatively narrow scope for variation within each game. Although a reasonable sample of 40 participants was recruited for Phase 1, a small group of 16 participants participated in Phase 2 of the study. Longer-term acceptability and feasibility was therefore only evaluated in eight individuals. Investigation of efficacy was not the primary focus of the study and was limited by sample size, participant heterogeneity, use of a control group who did not receive an equivalent amount of additional therapy and participant engagement in concurrent therapy.

This study provides important information on the feasibility of using game-based treatment approaches in a rehabilitation setting in people with a range of post-stroke deficits. This information may assist research and development of new stroke rehabilitation-specific games. In future studies the recruitment of a larger sample of participants and testing against an activity-matched control group should be considered. We recommend that the games used should promote functional movements and provide an optimal level of challenge that is tailored to the individual. Our findings suggest that the difficulty levels of the games may need to be extended as to suit individuals who are either lower or higher functioning. Studies should investigate which activities are most suited to particular sub-groups of participants and what outcome measures will best reflect any functional improvements made. Important aspects of feasibility, including participant acceptability, motivation, adherence, and safety, should continue to be explored. The evaluation of longer term and home-based use of this type of intervention is also critical to adequately inform feasibility, efficacy and cost-effectiveness.

## Conclusions

Training using interactive motion-controlled games appears largely feasible and acceptable for use across post-stroke individuals with a broad range of abilities. However, modifications to this approach are suggested and future intervention studies with larger samples are recommended to further explore longer-term feasibility, safety and clinical efficacy for improving physical outcomes in people with stroke.

## Abbreviations

3D: Three-dimensional
FIM: Functional independence measure
VAS: Visual analogue scale
ANOVA: Analysis of variance
SD: Standard deviation
IQR: Interquartile range
